# *Pseudomonas* spp. can help plants face climate change

**DOI:** 10.3389/fmicb.2023.1198131

**Published:** 2023-06-23

**Authors:** Antoine Zboralski, Martin Filion

**Affiliations:** Agriculture and Agri-Food Canada, Saint-Jean-sur-Richelieu Research and Development Centre, Saint-Jean-sur-Richelieu, QC, Canada

**Keywords:** *Pseudomonas*, plants, climate change, abiotic stress, salt, drought, heat, flood

## Abstract

Climate change is increasingly affecting agriculture through droughts, high salinity in soils, heatwaves, and floodings, which put intense pressure on crops. This results in yield losses, leading to food insecurity in the most affected regions. Multiple plant-beneficial bacteria belonging to the genus *Pseudomonas* have been shown to improve plant tolerance to these stresses. Various mechanisms are involved, including alteration of the plant ethylene levels, direct phytohormone production, emission of volatile organic compounds, reinforcement of the root apoplast barriers, and exopolysaccharide biosynthesis. In this review, we summarize the effects of climate change-induced stresses on plants and detail the mechanisms used by plant-beneficial *Pseudomonas* strains to alleviate them. Recommendations are made to promote targeted research on the stress-alleviating potential of these bacteria.

## 1. Introduction

The surface temperature has increased by 1.1°C globally and by 1.6°C on land since the pre-industrial era (IPCC, 2021). This rise in temperature is highly likely to continue, and even accelerate in the coming decades, leading to more intense and frequent droughts, extremes of heat, and other major weather events (IPCC, 2021, 2022; Lesk et al., 2022). While mitigation of climate change has been deemed critical to hamper this global threat, adaptation is also required to minimize the vulnerability of the affected agroecosystems and of the societies that rely on them (IPCC, 2022).

Agriculture is increasingly affected worldwide by this changing climate, notably through droughts, heatwaves, increased soil salinity, and floods (Tomaz et al., 2020; Bezner Kerr et al., 2022). These extreme events generate stress in plants, i.e., changes in growth conditions altering or even disrupting the plant homeostasis (Shulaev et al., 2008). Agricultural yields are already affected, with impacts ranging from slowing to halting yield growth in regions such as Australia and Southern Europe. A 10–20% decrease in yields has even been reported for some crops in Western Africa (Bezner Kerr et al., 2022).

Plants may be able to acclimate and adapt to some extent to climate change with the help of their microbiome (Trivedi et al., 2022). Indeed, plants are closely associated with a myriad of microorganisms, including protists, fungi, and bacteria, which form its microbiome. This microbiome helps plants acquire nutrients, enhance growth-related physiological processes, promote defense against plant pathogens, and alleviate abiotic stresses (Rubin et al., 2017; Trivedi et al., 2020). These microbes mainly colonize two plant-dependent compartments: the phyllosphere and the rhizosphere, which refer to the external surface of leaves and to the volume of soil influenced by the roots, respectively (Zboralski et al., 2023).

Bacteria belonging to the genus *Pseudomonas* are often core members of the phyllosphere and rhizosphere microbiome, competitively colonizing these compartments and thriving in them (Trivedi et al., 2020; Zboralski and Filion, 2020). These bacteria are rod-shaped, Gram-negative, motile, non-sporulating, and mostly aerobic organisms (Palleroni, 2005). On average, the genomes of *Pseudomonas* type strains consist of 5.6 ± 1.0 Mb and contain 5,260 ± 928 genes (Hesse et al., 2018). These bacteria can also carry plasmids, even if they are not commonly encountered (Silby et al., 2011). More than 300 species of *Pseudomonas* have been described so far according to the List of Prokaryotic names with Standing in Nomenclature (Parte et al., 2020).

Numerous *Pseudomonas* strains have been studied over the last decades for their biocontrol and plant growth promotion abilities (Weller, 2007; Mercado-Blanco, 2015). These plant-beneficial strains produce a multitude of secondary metabolites, including cyclic lipopeptides, antibiotics, siderophores, effectors, and plant hormones (Gross and Loper, 2009; Ghequire and De Mot, 2014; Götze and Stallforth, 2020; Biessy and Filion, 2021). These compounds mediate several direct and indirect plant-beneficial effects of *Pseudomonas* spp., for instance through the modulation of plant hormone levels and improved nutrient availability in the soil, or through the inhibition of plant pathogens and improved plant resistance to infections.

*Pseudomonas* strains have received increasing attention for their potential to relieve plants from environmental stresses (Supplementary Tables S1, S2; Rajkumar et al., 2017). Whether they are used as single-strain inoculants or as members of microbial consortia, *Pseudomonas* spp. show promise in alleviating stresses in crop plants exacerbated by climate change. Therefore, and given their biocontrol capabilities, the use of *Pseudomonas* spp. as bioinoculants in agriculture could actively contribute to reducing input costs, chemical contamination, pesticide use and exposure, and improving resilience at multiple levels.

How do the climate-related stresses impact plants? What are the mechanisms involved in the plant stress-alleviating effects of *Pseudomonas* spp.? What are the pending issues that need to be addressed by researchers to better use *Pseudomonas* strains in efforts to adapt agriculture to climate change and extreme weather events? Through this review, we aim to answer these questions and contribute to supporting the work in progress on the development of bioinoculants to better adapt agriculture to climate change.

## 2. Abiotic stresses exacerbated by climate change affect plants in different ways

### 2.1. Drought and high salinity create osmotic stress

Stresses caused by drought and high salinity often originate from distinct causes but lead to similar impacts for the plant (Forni et al., 2017).

**Figure 1:**
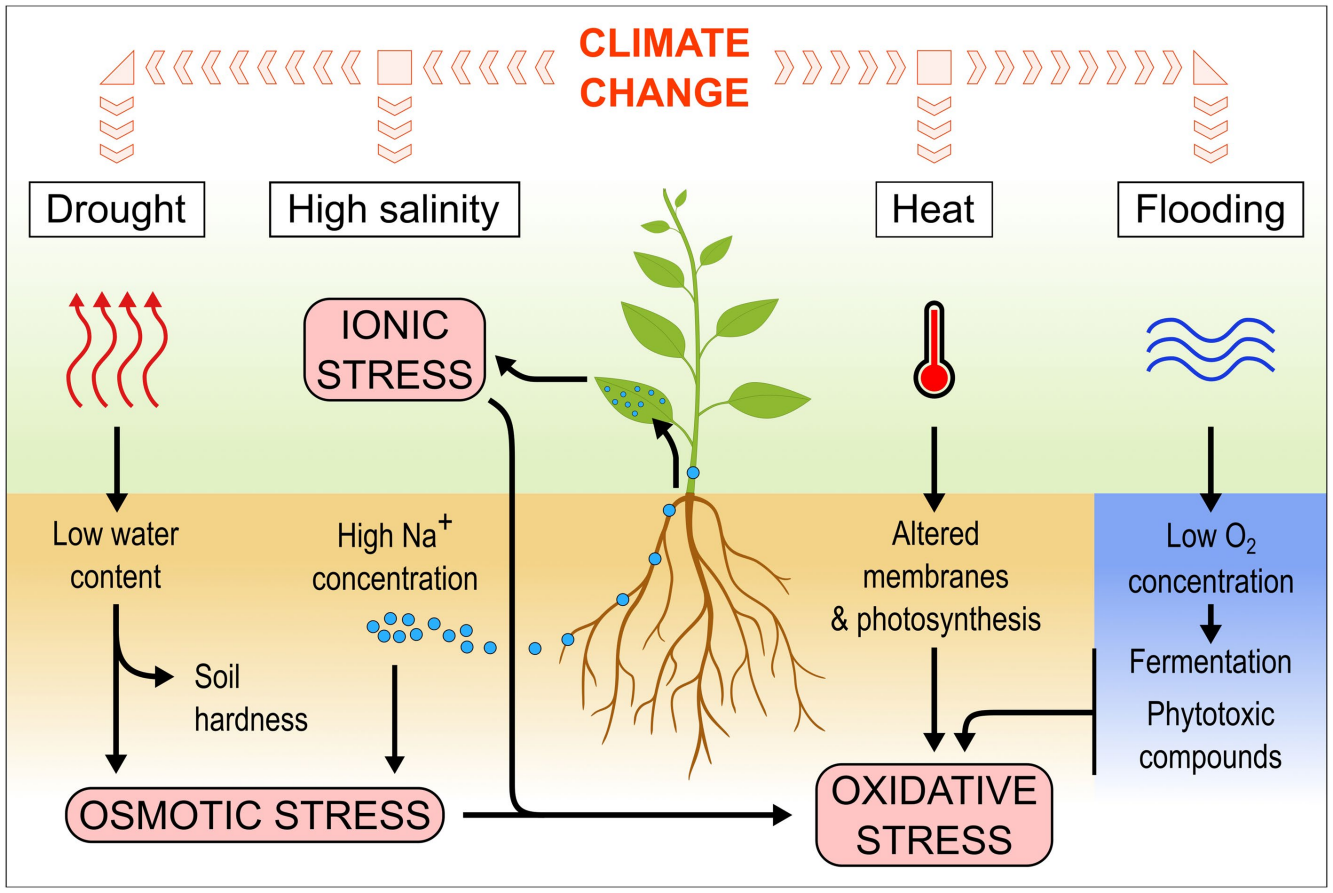
Climate-related stresses affect plants in different ways and lead to various cellular stresses.

Drought is usually defined as a deficiency in rainfall resulting in water shortage (Wilhite and Glantz, 1985). In agriculture, drought can be more specifically defined as insufficient rainfall for a given period of time whereby crop water requirements can no longer be met by the available water supply (Kebede et al., 2019). Such a lack in water availability decreases the soil water potential, creating osmotic stress for the plant (Figure 1). It also leads to a higher soil hardness, which reduces soil penetrability for roots (Colebrook et al., 2014). Drought is considered the main cause of losses in agriculture (FAO, 2021b).

Salinity stress in plants is caused by an excess of soluble salts in the soil, especially sodium chloride (Munns and Tester, 2008). Such excess in soluble salts can originate from low drainage, saltwater intrusion in coastal regions due to the rising of sea levels, or irrigation and land clearing raising the water table (Munns and Tester, 2008). Worldwide, at least 4.4% of topsoil (0–30 cm) and more than 8.7% of subsoil (30–100 cm) land area is affected by salt (FAO, 2021a). This area is likely to expand and soil salinity to increase in several regions because of climate change (Hassani et al., 2021). High salt concentrations in the soil decrease the soil water potential, making it more difficult for the plant to take up water, leading to osmotic stress, similar to drought conditions (Figure 1). Additionally, the plant takes up an excess of salts, which accumulates in the leaves to toxic levels, leading to ionic stress and impairing photosynthetic capacity (Munns and Tester, 2008).

Leaf growth tends to be more affected by osmotic stress than root growth as the plant saves water by limiting evapotranspiration through stomatal closure and keeps exploring the soil through its roots in search of the valuable molecule (Forni et al., 2017). Drought can also accelerate the crop cycle by triggering the reproductive phase earlier, leading to lower yields (Desclaux and Roumet, 1996). If drought or high salt concentration in soils persist, cells may lose membrane integrity and water, impairing photosynthesis and generating reactive oxygen species (ROS), eventually leading to plant death (Forni et al., 2017; Shahid et al., 2020). Plants respond to drought and high salinity through the production of ROS-scavenging enzymes, osmolytes, and secondary compounds like anthocyanins and phenolics, which help them acclimate to the resulting stresses (Forni et al., 2017).

### 2.2. Heat stress produces a physiological shock

Heat stress can be defined as an increase in temperature above a given threshold over a period of time sufficient to induce irreversible damages to plants, reducing yields (Wahid et al., 2007). It is difficult to provide the reader with a specific threshold or even a range of temperatures above which heat stress occurs, because plant species and cultivars all have different optimal growth temperatures and sensitivity to temperature variation (Hatfield and Prueger, 2015). Also, the effects of heat stress on yields are not linear: each additional degree above the optimal growth temperature for a given crop results in a greater yield loss than the previous degree (Schlenker and Roberts, 2009). Although all the developmental stages of plants are vulnerable to heat stress, the extent of this vulnerability varies according to the stage (Jagadish et al., 2021). The flowering, gametogenesis, and pollination stages are especially sensitive to heat (Hatfield and Prueger, 2015; Jagadish et al., 2021). Heat affects membrane and cuticle integrity, and enzyme activity, leading to impaired photosynthesis as well as increased respiration and oxidative stress (Figure 1; López et al., 2022).

When water is available in the soil, plants can use it to cool down the leaves and limit the effects of heat (López et al., 2022). However, when drought and heat combine, which is expected to be increasingly common due to climate change (Cohen et al., 2021), leaf temperature can increase by 5°C to 10°C, causing severe damage to the whole plant and resulting in a sharp decline in crop yield (López et al., 2022). Plants can also acclimate to some extent to heat using mechanisms that resemble those used to cope with drought or high salinity conditions. They accumulate osmolytes such as polyol, proline, and ammonium compounds, produce secondary metabolites like isoprenoids or carotenoids, activate detoxification systems against ROS, and synthesize heat shock proteins to protect other proteins from denaturation (Wahid et al., 2007).

### 2.3. Flooding stress drastically reduces oxygen availability

Flooding can be broadly defined as excessively wet conditions in which water occupies volumes around roots and/or shoots that are normally filled with air (Sasidharan et al., 2017). It especially encompasses two phenomena: waterlogging and submergence. Waterlogging is a soil condition whereby excess water inhibits gas exchange with the atmosphere at the root level only (McFarlane et al., 1989; Sasidharan et al., 2017). In practice, waterlogging is happening when the water table is less than 30 cm below the soil surface, or when more than 90% of the soil pores are filled with water (Shaw and Meyer, 2015). Submergence occurs when free-standing water is above the soil surface, partially or completely submerging the plant (McFarlane et al., 1989; Sasidharan et al., 2017). Worldwide, flooding is considered the second most impactful disaster in agriculture, after drought (FAO, 2021b). Most crops are not adapted to grow in water-saturated soils, which can lead to severe yield losses depending on the crop, soil type, and the duration of the flood (Zhou, 2010). In coastal areas, flooding stress can combine with salt stress, exacerbating yield losses (Zhou, 2010).

Under water-saturated conditions, access to oxygen is limited because of its poor diffusion in water compared to air (Watanabe et al., 2013). The low amount of available oxygen is quickly consumed by the roots and microbes, leading to anoxic conditions and to the development of anaerobic microbes, which decrease the redox potential in the flooded soil (Figure 1; Laanbroek, 1990; Watanabe et al., 2013). Under prolonged flooding, phytotoxic reduced forms of inorganic compounds accumulate in the soil, such as sulfides (Singh et al., 2018). Without oxygen, plants are unable to perform respiration and use fermentation instead to generate usable forms of energy (Perata and Alpi, 1993). If the plants are entirely submerged, they receive less light, impacting photosynthesis and further reducing oxygen availability (Loreti et al., 2016). They actively degrade starch to maintain glycolysis and survive, but growth is generally strongly reduced, leading to yield losses (Loreti et al., 2016, 2018).

## 3. *Pseudomonas* spp. directly affect phytohormone levels to help plants cope with climate-related stresses

The mechanisms used by *Pseudomonas* spp. to help plants cope with abiotic stresses intensified by climate change are often not specific to a single stress and rather help the plant grow under various stressful conditions. They especially consist in producing compounds directly affecting the plant hormone levels, notably ethylene, auxin, gibberellins, and cytokinins.

### 3.1. Reduction of ethylene levels in plants

The reduction of plant ethylene levels is certainly one of the most studied bacteria-mediated plant stress relief mechanisms. Ethylene is a chemically simple gaseous plant hormone that is particularly well known for its role in fruit ripening (Barry and Giovannoni, 2007). It also plays a central role in growth and response to biotic and abiotic stresses (Dubois et al., 2018). Ethylene concentration increases when plants face drought, heat, high salt, and flooding, resulting in reduced growth (Dubois et al., 2018; Pattyn et al., 2021).

**Figure 2:**
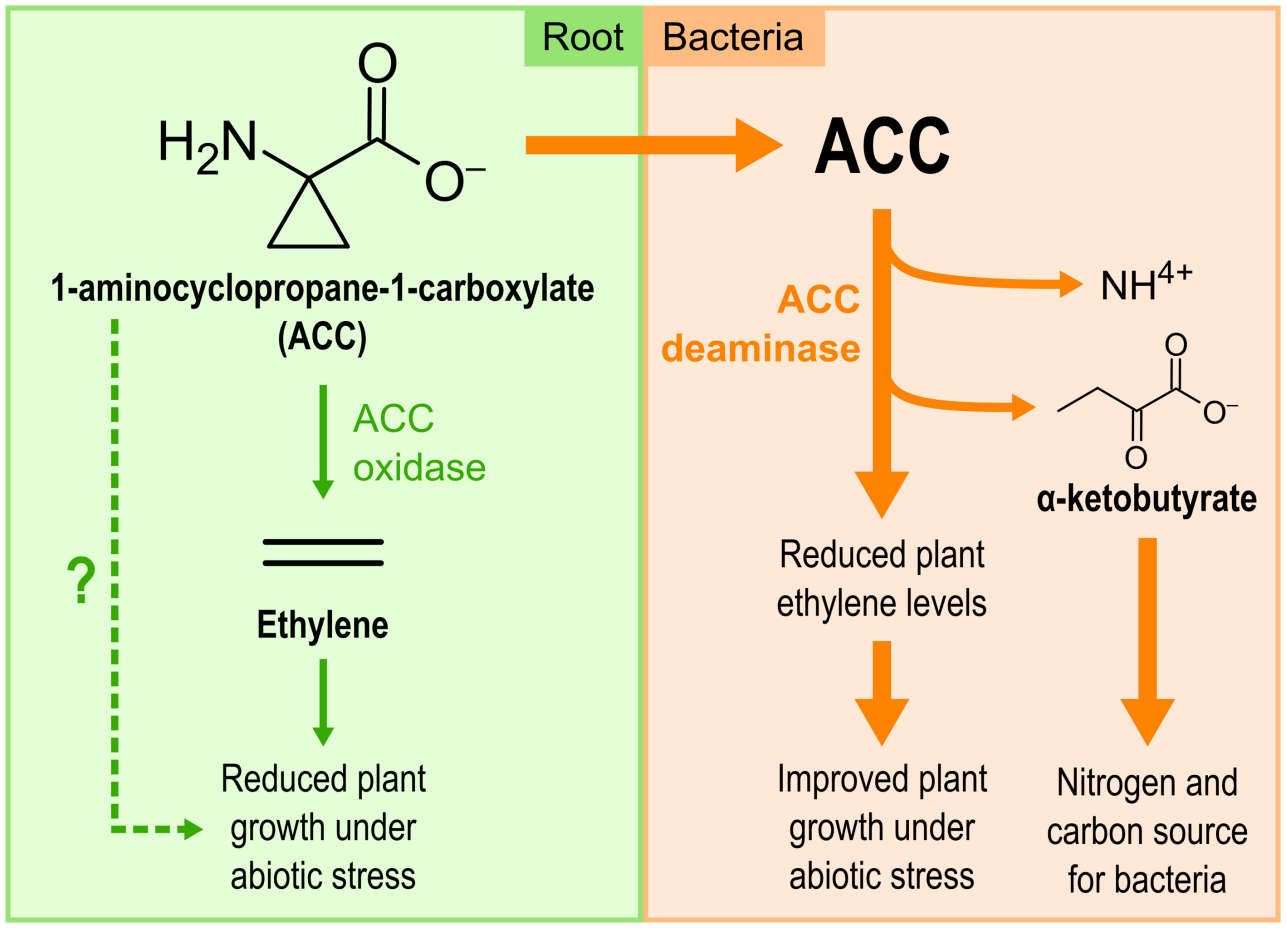
The 1-aminocyclopropane-1-carboxylate (ACC) deaminase produced by *Pseudomonas* spp. reduces ethylene levels in plants under climate-related abiotic stresses.

Some bacteria, including multiple *Pseudomonas* strains belonging to diverse species, can lower the plant ethylene levels by producing the AcdS enzyme, a cytoplasmic deaminase that degrades the direct precursor of ethylene, 1-aminocyclopropane-1-carboxylate (ACC), into ammonium and α-ketobutyrate (Figure 2; Nascimento et al., 2014; Glick and Nascimento, 2021). ACC itself has recently been proposed as a signal molecule for plants, involved in various plant processes such as pathogen interactions and stress response (Polko and Kieber, 2019). Therefore, the activity of AcdS decreases the concentration of not only one, but two signaling molecules involved in plant stress responses. This leads to enhanced plant growth under stress conditions and generates usable sources of nitrogen and carbon for the bacteria (Glick and Nascimento, 2021).

The effect of the ACC deaminase produced by *Pseudomonas* strains on plants under stress conditions has been demonstrated in several plant species using reverse genetics approaches, especially in canola, cucumber, and tomato under salt stress, and in tomato under flooding stress (Glick and Nascimento, 2021). Some research teams also used a transgenic approach, transferring a *Pseudomonas acdS* gene directly into the genome of tomato and *Arabidopsis thaliana*, which then became more tolerant to a flooding period of 5–9 days (Grichko and Glick, 2001; Jung et al., 2018). These two approaches clearly demonstrated the active role played by ACC deaminase in plant stress alleviation.

### 3.2. Direct biosynthesis of phytohormones

Some *Pseudomonas* spp. have been shown to directly produce phytohormones, especially IAA, gibberellins, and cytokinins, while promoting plant growth under abiotic stress conditions at the same time (García de Salamone et al., 2001; Egamberdieva, 2009; Kang et al., 2014; Spaepen, 2015; Mekureyaw et al., 2022; Yasmin et al., 2022).

#### 3.2.1. Auxin biosynthesis

**Figure 3:**
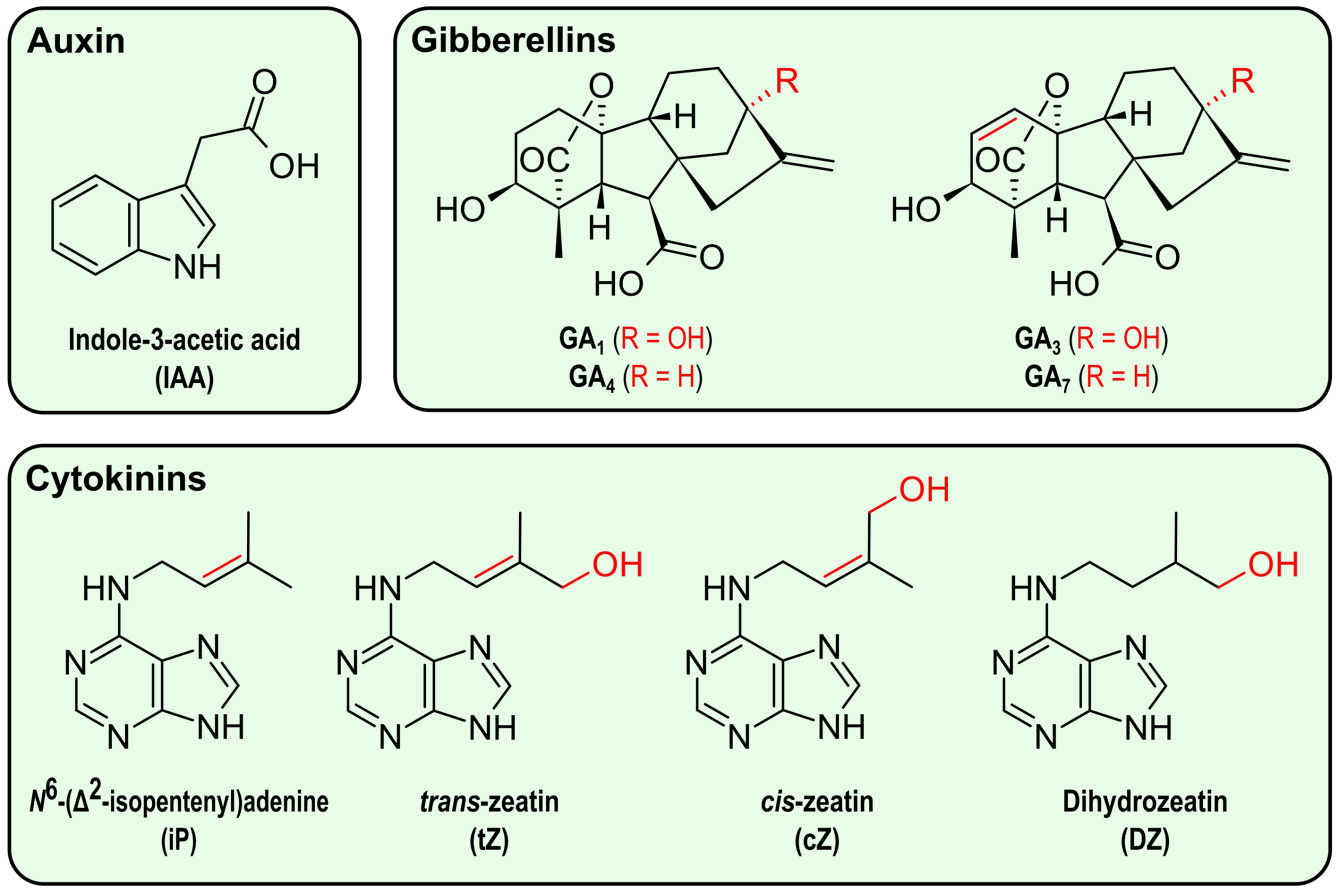
Structure of the main phytohormones produced by bacteria. Elements in red represent variations between compounds in the same category.

Auxin—from the Greek word “auxein,” “to grow”—usually refers to indole-3-acetic acid (IAA; Figure 3), the main plant auxinic compound (Enders and Strader, 2015). Its role in plants has historically been demonstrated in cell elongation and apical dominance, but this phytohormone is also involved in many other growth and development processes in plants, if not all (Zhao, 2010; Leftley et al., 2021). For the last decade, its role in the plant response to abiotic stresses has been further explored (Sharma et al., 2015). Auxin has notably been shown to contribute to the optimization of the root system architecture under environmental stress, especially osmotic stress (Leftley et al., 2021).

Many *Pseudomonas* spp. have been shown to produce IAA, and in some cases, to improve plant tolerance to climate-related stresses. For instance, several strains known to produce IAA can reduce the effect of high salinity on germination and seedling growth in wheat and cotton (Egamberdieva, 2009; Egamberdieva et al., 2015). Under drought conditions, other IAA-producing *Pseudomonas* strain were able to improve growth in different plant species, including wheat, jujube, and *A. thaliana* (Chandra et al., 2018; Raheem et al., 2018; Zhang et al., 2020; Yasmin et al., 2022). Heat stress could also be alleviated in wheat using an IAA-producing, thermotolerant strain (Ali Shaik et al., 2011). Using IAA-deficient mutants, some research groups demonstrated that IAA produced by *Pseudomonas* strains played a substantial role in plant growth promotion under standard growth conditions (Patten and Glick, 2002; Ul Hassan and Bano, 2019). However, to our knowledge, such a clear causal link remains to be demonstrated under climate-related stress conditions. Interestingly, external IAA application on a strain belonging to another *Proteobacteria* genus, *Bradyrhizobium*, improves its tolerance to various abiotic stresses such as osmotic stress (Duca and Glick, 2020). IAA might also contribute to stress tolerance in *Pseudomonas* strains.

Three main IAA biosynthetic pathways have been uncovered in plant-beneficial *Pseudomonas* spp. and are all tryptophan-dependent (Duca et al., 2014, 2018). The indole-3-acetamide (IAM) pathway is mediated by enzymes encoded by the *iaa* gene cluster (Gross and Loper, 2009). Many *Pseudomonas* strains carry this cluster in their genome (Spaepen and Vanderleyden, 2011; Loper et al., 2012; Biessy et al., 2019). The indole-3-pyruvic acid (IPA) pathway, including the shortcut enabled by a tryptophan side-chain oxidase, has been found in *Pseudomonas* spp., but the genes involved have not yet been identified in strains belonging to this genus (Duca et al., 2014; Duca and Glick, 2020). The *ipdC* gene, encoding a key enzyme in the IPA pathway, has been detected in several bacterial genera (Duca et al., 2014). This gene had been identified in a single *Pseudomonas* strain, *P. putida* GR12-2 (Patten and Glick, 2002). However, the reported sequence turned out to originate from a strain belonging to another *Gammaproteobacteria* genus, *Enterobacter* (Gross and Loper, 2009). Moreover, BLASTn and BLASTp searches using *ipdC* sequences from various genera did not yield any significant results in the *Pseudomonas* genus, suggesting the implication of genes distinct from *ipdC* in this pathway. The third IAA pathway identified in *Pseudomonas* spp. is the indole-acetaldoxime/indole-3-acetonitrile (IAOx/IAN) pathway (Duca et al., 2014). The genes encoding the enzymes involved in this pathway have almost all been identified in *P. putida* UW4: *phe*, *nit*, *nthA*, *nthB*, and *ami* (Duca et al., 2018). Few other *Pseudomonas* strains have been shown to harbor homologous genes in their genome (Duca et al., 2014). Only the gene that encodes the enzyme mediating the first step of the pathway remains to be identified, along with the protein itself (Duca et al., 2018). All three pathways can be found in a single strain, for example in *P. putida* UW4, in which they are interrelated (Duca et al., 2018).

Some *Pseudomonas* strains harbor the *iac* gene cluster, responsible for IAA catabolism, sometimes harboring the IAA biosynthetic cluster as well (Loper et al., 2012; Biessy et al., 2019; Zboralski et al., 2022). This cluster allows its carrier strain to use IAA as the sole carbon and energy source (Leveau and Gerards, 2008). Since the optimal IAA concentration to favor plant growth is narrow (Persello-Cartieaux et al., 2003), plant-beneficial *Pseudomonas* must finely tune the external IAA concentration to benefit from plant growth. This catabolic pathway may contribute to such regulation. Under climate-related stresses, this balance between IAA biosynthesis and degradation in plant-beneficial *Pseudomonas* spp. and its role in stress-alleviation remains to be investigated.

#### 3.2.2. Gibberellins biosynthesis

Gibberellins are tetracyclic diterpenoid carboxylic acids whose name originates from the fungus *Gibberella fujikuroi* (now *Fusarium fujikuroi*), a rice pathogen from which some gibberellins were first isolated for their role in shoot elongation (Hedden and Sponsel, 2015). These hormones control transitions between plant stages as well as cell division and elongation (Colebrook et al., 2014). The main bioactive gibberellins are GA_1_, GA_3_, GA_4_, and GA_7_ (Figure 3), with GA_1_ and GA_4_ being the most common ones in plants (Binenbaum et al., 2018). Under stress conditions such as flooding, drought and high salinity, the plant gibberellin signaling pathway is usually inhibited, allowing the plant to adapt its growth to the new conditions and prevent ROS accumulation (Achard et al., 2008; Colebrook et al., 2014).

Gibberellins biosynthesis has been reported in some plant-beneficial *Pseudomonas* strains (Kang et al., 2014, 2020; Pandya and Desai, 2014; Sharma et al., 2018; Yasmin et al., 2022). However, the methods used to detect and quantify gibberellins differ greatly, which can lead to unreliable claims (Bottini et al., 2004). These methods range from basic spectrophotometric approaches (Pandya and Desai, 2014; Sharma et al., 2018) to more robust methods involving analytical chemistry tools, including gas chromatography–mass spectrometry (Kang et al., 2014, 2019, 2020) and high-performance liquid chromatography (García de Salamone et al., 2001; Yasmin et al., 2022). Analytical approaches allowed to accurately detect different bioactive gibberellins in *Pseudomonas* strains that were able to promote the growth of soybean, *A. thaliana*, lettuce, and Chinese cabbage, in some cases under high salinity and drought conditions (Kang et al., 2014, 2019; Adhikari et al., 2020; Yasmin et al., 2022).

The gibberellin biosynthetic pathway has been elucidated recently in bacteria compared to its distinct counterparts in plants and fungi (Nett et al., 2017). The biosynthetic operon has been well described in rhizobia and notably contains three cytochrome P450 monooxygenases, which are each involved in multiple steps leading to the synthesis of GA_9_ (Nett et al., 2017). The entire operon has been identified in two *Pseudomonas* strains only, *P. psychrotolerans* NS274 and *P. psychrotolerans* RSA46 (Nagel et al., 2018). Their operon is quite distant from other bacterial ones and include a fourth cytochrome P450 monooxygenase that is known to enable the conversion of GA_9_ to the bioactive compound GA_4_ (Nagel et al., 2018). These strains were both isolated from rice seeds and have not been assessed yet for potential plant-beneficial effects and gibberellin production (Midha et al., 2016). Interestingly, they seem to be the only known bacterial strains carrying this operon that are neither rhizobia nor known pathogens (Nagel et al., 2018). However, the nature of their potential relationship with plants remains to be assessed. Considering that hundreds of high-quality *Pseudomonas* genomes are now available and that several *Pseudomonas* strains are known to produce gibberellins, it is surprising that only two strains have been shown to display the biosynthetic operon in their genome. Sequencing the genomes of the strains that are already known to produce gibberellins would likely help identify the associated biosynthetic pathway. Also, this biosynthetic pathway may be different than the only one described to date in other bacteria (Nett et al., 2017). More research efforts are needed to elucidate this enigma and build gibberellin-defective mutants. These mutants will be needed to assess the exact role *Pseudomonas*-produced gibberellins may play in the alleviation of climate-related stresses in plants.

#### 3.2.3. Cytokinins biosynthesis

Cytokinins are a family of adenine derivatives initially described for their roles in cell division, from which their name is derived, and growth (Skoog and Armstrong, 1970). They have later been characterized for their involvement in the plasticity of the root system architecture under drought conditions, in sodium exclusion under high salinity conditions, as well as in the plant response to heat stress (Cortleven et al., 2019; Li et al., 2022). Different cytokinins have been found in plants, but the main ones are isoprenoid cytokinins, including *N*^6^-(Δ^2^-isopentenyl)-adenine (iP), *trans*-zeatin (tZ), *cis*-zeatin (cZ), and dihydrozeatin (DZ) (Figure 3; Sakakibara, 2006; Cortleven et al., 2019).

Cytokinin production has long been known to be associated with some plant-pathogenic *Pseudomonas* strains and has also been described in some plant-beneficial ones (García de Salamone et al., 2001; Pérez-Martínez et al., 2008; Großkinsky et al., 2016). The tZ-producing strain *P. putida* AKMP7 has been shown to alleviate heat stress in wheat (Ali Shaik et al., 2011; Raja Gopalan et al., 2022). Another cytokinin-producing *Pseudomonas* strain, *Pseudomonas* sp. G20-18, was shown to increase cytokinin concentrations in the rhizosphere of canola and to prime the tomato stress response to drought (Pallai et al., 2012; Mekureyaw et al., 2022). Interestingly, cytokinin biosynthesis was also demonstrated to induce plant defenses against *P. syringae* pv. *tomato* DC3000 when this pathogen and G20-18 were infiltrated in *A. thaliana* leaves (Großkinsky et al., 2016).

Two distinct cytokinin biosynthetic pathways have been described in bacteria: *de novo* biosynthesis from adenosine monophosphate initiated by an adenylate isopentenyl transferase (IPT), and synthesis from a modified adenosine in specific tRNA catalyzed by a tRNA-IPT (Großkinsky et al., 2016; Frébortová and Frébort, 2021). Wei et al. recently published a comparative analysis of the adenylate IPTs in plant-beneficial and plant-pathogenic bacteria (Wei et al., 2023). They showed that the genomes of plant-beneficial bacteria tended more often to harbor genes related to cytokinin degradation and metabolism in the vicinity of adenylate IPT than genomes of pathogens, which contained more adenylate IPT gene copies on average. This suggests that beneficial bacteria regulate cytokinin biosynthesis differently than pathogenic ones to optimize plant-beneficial effects (Wei et al., 2023). Homologs of the adenylate IPT gene were found in the genome of 90 bacteria, including only one *Pseudomonas* strain, *P. psychrotolerans* PRS08-11306 (Wei et al., 2023). This strain, which was isolated from rice seeds, was shown to improve rice growth but has not been tested yet for cytokinin production (Liu et al., 2017). The fact that only one *Pseudomonas* strain was shown to display an adenylate IPT gene in its genome suggests that the *de novo* cytokinin biosynthesis pathway may not be very common within this genus. The tRNA-IPT-encoding gene *miaA*, involved in the other cytokinin biosynthetic pathway, is found in almost all bacterial species, including *Pseudomonas* spp., probably because of its role in tRNA modification and in translation (Carpentier et al., 2020; Frébortová and Frébort, 2021; Nielsen et al., 2021; Wei et al., 2023). Also, mutations in *miaA* often result in pleiotropic effects (Gibb et al., 2020). This makes it difficult to study the effect of cytokinin-deficient mutants on plants, although a viable *miaA*-defective mutant was successfully engineered in *Pseudomonas* sp. G20-18 (Großkinsky et al., 2016). In the latter, the cytokinin production was not directly assessed, but its effect on cytokinin levels in *A. thaliana* was evaluated under standard conditions. It notably decreased the plant tZ and iP levels when compared to the wild type, suggesting a potentially lower cytokinin biosynthesis in this mutant (Großkinsky et al., 2016).

## 4. Volatile organic compounds mediate a remote stress-alleviating effect of *Pseudomonas* spp. in plants

Volatile organic compounds (VOCs) are low-molecular weight molecules displaying a high vapor pressure and a low boiling point, which allow them to diffuse easily in air and water (Netzker et al., 2020). Plant-beneficial *Pseudomonas* strains have been shown to produce a multitude of them, including alkenes, amides, aromatic compounds, esters, ketones, and sulphur compounds, which are involved in antimicrobial activity, elicitation of the plant immune responses, and plant growth promotion (Garbeva and Weisskopf, 2020; Netzker et al., 2020). However, only few of these compounds have been demonstrated to influence plants at concentrations observed *in vivo*, especially under abiotic stresses.

Several *Pseudomonas* strains can produce VOCs that directly help plants cope with drought or high salinity. *P. chlororaphis* subsp. *aureofaciens* O6 produces 2R,3R-butanediol, which induces systemic tolerance to drought and high salinity in *A. thaliana* by triggering stomatal closure (Cho et al., 2008, 2012). Another *Pseudomonas* strains, *P. pseudoalcaligenes* SMR-16, can improve maize growth under drought using volatile compounds (Yasmin et al., 2021). The emitted compounds were characterized and included 2R,3R-butanediol along with several others, such as dimethyl disulfide and 2-pentylfuran. The potential role played by each one in growth promotion was unfortunately not assessed. Finally, a research group identified a strain named *P. simiae* AU that was able to improve soybean growth under salt stress through a mix of volatile compounds, which remain to be characterized (Vaishnav et al., 2015). The volatile compounds produced by this strain induced the accumulation of proline and a reduction in the sodium content of roots, helping the plants cope with osmotic and ionic stress, respectively.

Other volatile compounds produced by plant-beneficial *Pseudomonas* strains have been shown to have a direct effect on the growth of different plant species, for instance 2-butanone, N,N-dimethyl-formamide, formamide, 1-hexanol, indole, 2-methyl-n-1-tridecene, and 13-tetradecadien-1-ol (Blom et al., 2011; Park et al., 2015; Zhou et al., 2016). To our knowledge, the potential effects of such compounds on plants under abiotic stresses have however not yet been investigated.

Curiously, after decades of research on the effects of microbial VOCs on plants, the mechanism(s) mediating their perception by plants remains mostly unknown (Bailly, 2020). Even though these molecules have been shown to display a plethora of plant-beneficial effects, no specific receptor proteins actually binding these microbial compounds have been found, even for the long-known 2,3-butanediol (Bailly, 2020; Weisskopf et al., 2021). Such a knowledge gap represents a major barrier to a better use of VOCs in the development of effective microbial inoculants. Bailly (2020) suggested a practical approach to lift this barrier, which consists in the identification of reliable traits induced by these compounds, the construction of a classification of these compounds based on their bioactivity, and large-scale transcriptomic and proteomic approaches to uncover how plants react to these molecules.

## 5. A *Pseudomonas* strain strengthens the root apoplast barriers, countering the effects of high salinity in plants

**Figure 4:**
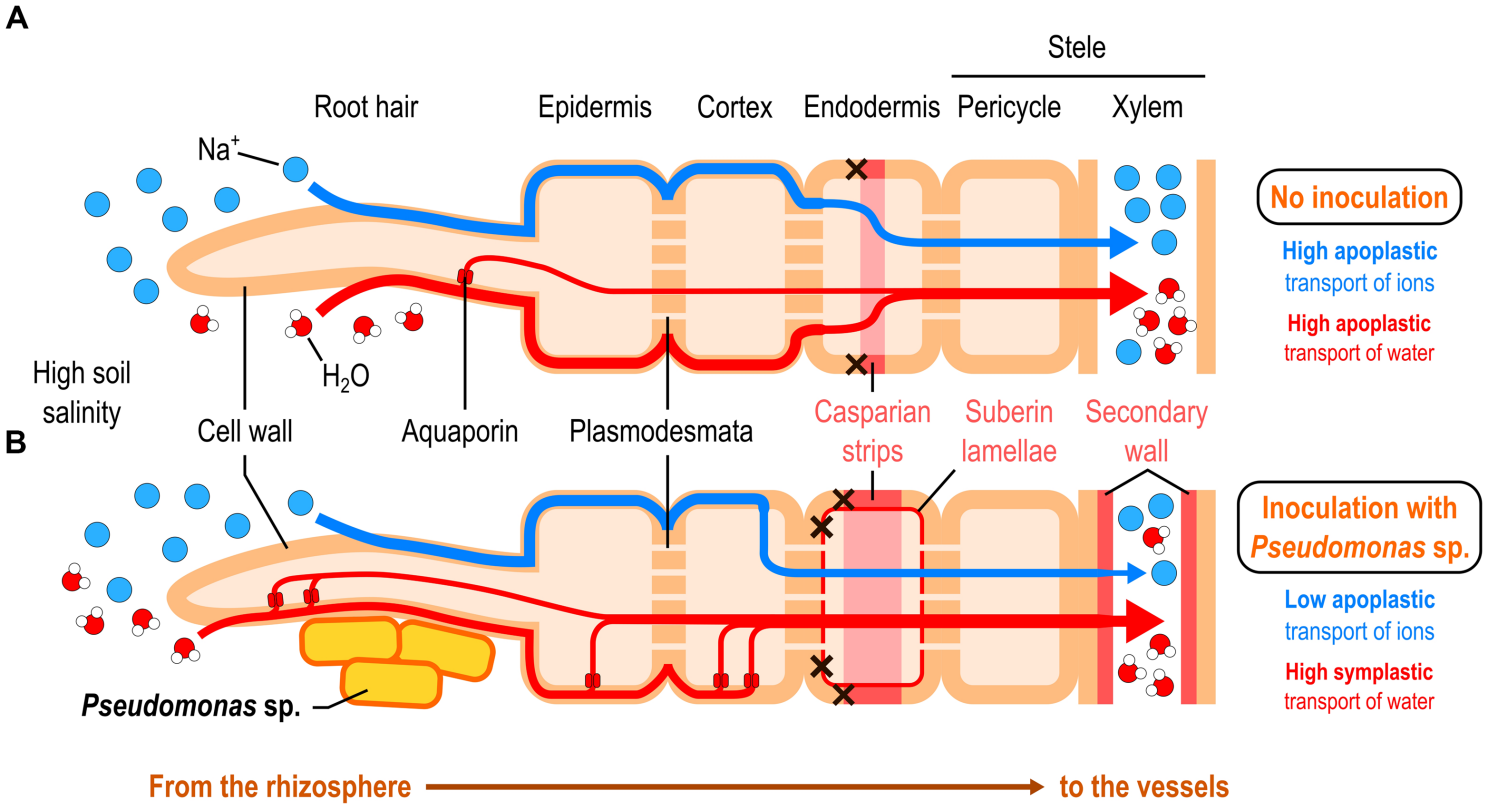
*Pseudomonas*-mediated reinforcement of the root apoplastic barriers under high soil salinity conditions. **(A)** Non-inoculated plants. **(B)** Plants inoculated with a *Pseudomonas* strain.

In two recent studies, a research group discovered that a *Pseudomonas* strain known as *P. mandelii* IB-Ki14 was able to help wheat and pea better tolerate salt in the soil by increasing the deposition of suberin and lignin in xylem cell walls, suberin lamellae, and Casparian strips (Figure 4; Martynenko et al., 2022, 2023). These structures are important apoplastic barriers in the root endodermis controlling water and mineral influx into the root stele. The authors hypothesized that the strengthening of these barriers would decrease the hydraulic conductance from the roots to the leaves, but this was not the case. Instead, they showed that bacterial inoculation led to a dramatic increase in the amount of aquaporins in the roots, especially around the epidermis and endodermis, under normal growing conditions (Arkhipova et al., 2022). Under high salinity conditions, aquaporin activity may compensate the loss of apoplastic permeability by improving symplastic transport of water, enabling the plant to better control water and sodium influx.

Accumulation of abscisic acid in plants, a phytohormone involved in the plant stress response, usually leads to these effects on apoplastic barriers under salt stress conditions (Martynenko et al., 2023). However, the authors showed that there was no such accumulation in pea plants inoculated with *P. mandelii* IB-Ki14, which led them to suggest that these salt stress-alleviating effects may be ABA-independent (Martynenko et al., 2023). However, *P. mandelii* IB-Ki14 was able to improve IAA levels in roots, which may mediate the observed effects. Further investigation is needed to decipher the underlying mechanism.

Interestingly, accumulation of suberin in roots of wetland plant species has been suspected to improve their tolerance to waterlogging by reducing root oxygen loss (Watanabe et al., 2013). Such an accumulation occurs in the root outer cell layers and not at the endodermis level. Therefore, these plant species have evolved flood-adapted root tissues that are central to flood tolerance, such as the aerenchyma. The *Pseudomonas*-induced suberin deposition in roots shown to help plants cope with high salinity might also contribute to flood tolerance. Such a hypothesis would have to be tested.

## 6. *Pseudomonas* spp. reduce oxidative stress in plants and also for their own survival

Under stress, plants accumulate reactive oxygen species (ROS), which can damage proteins, lipids, and nucleic acids, leading to impaired cellular processes and slower plant growth (Zhang et al., 2016; Smirnoff and Arnaud, 2019). Plants are however able to decrease ROS levels to some extent, through the production of antioxidant metabolites such as proline, ascorbate, and glutathione, or via the synthesis of antioxidant enzymes including superoxide dismutase, peroxidase, and catalase (Mittler, 2002; Zandi and Schnug, 2022). Several *Pseudomonas* strains have been shown to modulate the plant antioxidant activity under various stress conditions, leading to decreased oxidative stress. For instance, under high salinity conditions, *P. frederiksbergensis* OS261 was able to improve catalase activity and reduce superoxide dismutase activity in red pepper, while reducing ROS concentration overall (Chatterjee et al., 2017). Another strain, *P. putida* GAP-P45, decreased antioxidant enzyme activity and ROS levels in *A. thaliana* under osmotic stress, and increased proline turnover (Ghosh et al., 2017, 2018). The mechanisms mediating this modulation of the plant antioxidant machinery remain unknown (Rajkumar et al., 2017). However, another *Pseudomonas* strain, *P*. *guariconensis* MTCC5279 (formerly called *P. putida* MTCC5279), was shown to affect the regulation of antioxidant enzyme genes in chickpea during and following osmotic stress (Tiwari et al., 2016). This suggests an indirect effect of the *Pseudomonas* strain, potentially through phytohormone signaling pathways.

Plant-beneficial *Pseudomonas* must also produce their own antioxidant enzymes, such as peroxidases and superoxide dismutases, to colonize and survive in the plant vicinity (Kim and Park, 2014; Guan et al., 2017; Nikel et al., 2021). Plants generate ROS to regulate several basic processes including growth, development, and immunity (Liszkay et al., 2004; Baxter et al., 2014). Consequently, bacteria living close to plants, for example in the rhizosphere or the phyllosphere, are exposed to these reactive compounds, even more under climate-related stresses, which drive ROS accumulation in plants. The well-studied plant-beneficial strain *P. putida* KT2440 has recently been shown to display greatly improved root colonization abilities in alfalfa when overexpressing a specific peroxidase (Santamaría-Hernando et al., 2022). Its colonization levels were about 30 times higher in the roots and 900 higher when considering the root tip only, compared to the parent strain. Interestingly, this strain was also shown to improve tolerance to high salinity in soybean and maize, and to drought in tomato (Costa-Gutierrez et al., 2020a,b; Saglam et al., 2022). Strains with such enhanced antioxidant activity may then be more effective to improve plant tolerance to climate-related stresses. This remains to be assessed but antioxidant activity could be a relevant criterion when screening for plant-beneficial *Pseudomonas* strains.

## 7. Exopolysaccharide production by *Pseudomonas* spp. improves rhizosphere soil structure and water content under climate-related stresses

Many plant-beneficial *Pseudomonas* spp. form biofilms, which are aggregates of bacteria attached to a surface and/or to each other, embedded in a matrix of highly hydrated polymeric compounds that they secrete (Flemming et al., 2016; Zboralski and Filion, 2020). These compounds include proteins, lipids, extracellular DNA, and exopolysaccharides (Flemming et al., 2016). Biofilms improve the tolerance of their producing bacteria to various stresses, such as osmotic and oxidative stress, and enable them to reach cell densities needed to produce plant-beneficial secondary metabolites (Danhorn and Fuqua, 2007; Flemming et al., 2016; Svenningsen et al., 2018). The role played by biofilm formation, and especially by exopolysaccharides, in the alleviation of climate-related stresses, has been investigated in several *Pseudomonas* strains. In this regard, strain *P. putida* GAP-P45 has notably been studied (Sandhya et al., 2009, 2010a,b; Sandhya and Ali, 2015). This strain produces relatively high amounts of exopolysaccharides under drought and high salinity conditions (Sandhya et al., 2010b; Sandhya and Ali, 2015). It was shown to alleviate osmotic stress in sunflower and maize and to increase the amount of exopolysaccharides in the rhizosphere, along with improving soil aggregate stability and leaf relative water content (Sandhya et al., 2009, 2010a). Another team investigated the effects of high salinity in soybean and maize in three mutants of *P. putida* KT2440. These mutants were impaired in *lapA*, *lapF*, or both genes, which are necessary for cell-surface and cell–cell attachment, respectively (Costa-Gutierrez et al., 2020b). They were not able to produce mature biofilms but were shown to overproduce exopolysaccharides and to display exopolysaccharide profiles distinct from the wild type, potentially to compensate for biofilm defects (Martínez-Gil et al., 2013). Inoculation with the double *lapA*-*lapF* mutant better increased the fresh and dry weight of maize plants than inoculation with the wild type, which already improved plant growth. Exopolysaccharides may then help the bacteria cope with osmotic and ionic stress in soil, but may also directly help the plant by improving water retention in the rhizosphere and by binding to cations, which limit their penetration into the plant (Costa-Gutierrez et al., 2020b). Nonetheless, characterization of the exopolysaccharides produced by rhizosphere *Pseudomonas* strains is lacking (Zboralski and Filion, 2020). This information could contribute to a better understanding their role in the alleviation of abiotic stresses in plants.

## 8. Conclusion and research perspectives

**Figure 5:**
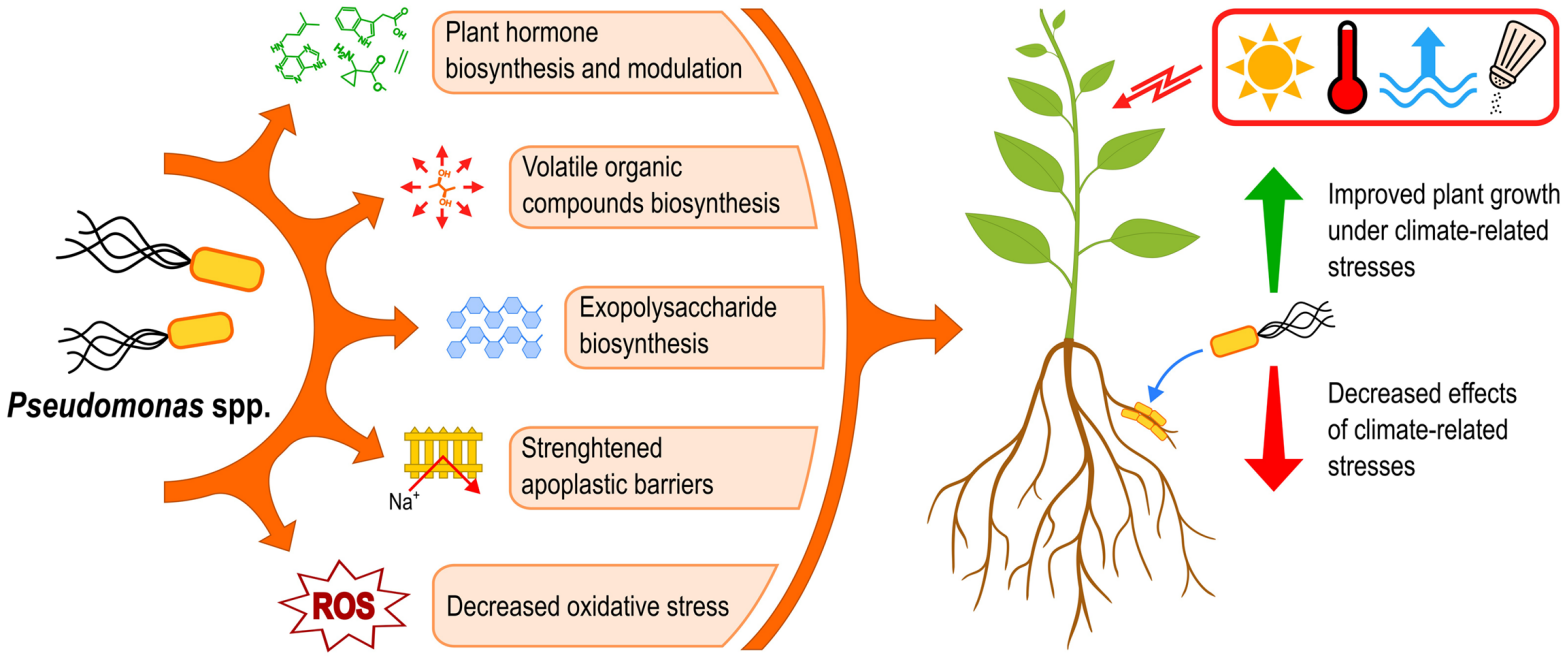
Plant-beneficial *Pseudomonas* strains use a variety of mechanisms to improve the plant tolerance to climate-related stresses. ROS, reactive oxygen species.

Plants are facing increasing pressure from climate change globally, notably through drought, high salinity, heat, and flooding. The effects of these stressful conditions on plants are diverse and can combine and amplify each other. However, many bacterial strains belonging to the genus *Pseudomonas* have been shown to improve plant growth under such stresses, by producing plant hormones, emitting volatile compounds, reinforcing the root apoplast barriers, mitigating oxidative stress, and excreting exopolysaccharides (Figure 5). These mechanisms can decrease the effects of climate-related stresses on plants and directly promote growth at the same time, improving yields overall under such abiotic stresses. The great diversity and versatility of plant-beneficial *Pseudomonas* strains could thus offer many opportunities to successfully develop *Pseudomonas*-based bioinoculants able to counter the effects of climate-related stresses. However, many gaps in knowledge must be addressed to achieve this goal. Some perspectives and recommendations are presented hereinafter to overcome these obstacles.

### 8.1. Sequence the genome of promising strains

Many *Pseudomonas* strains of interest for abiotic stress alleviation, often studied for many years, do not have their entire genome sequenced. Such information can be instrumental in understanding the molecular mechanisms of stress alleviation, while offering a reliable identification at the species level, much more reliable than 16S rRNA sequencing (Mulet et al., 2010; Garrido-Sanz et al., 2016; Zboralski et al., 2023). It especially enables transcriptional studies and site-directed mutagenesis, allowing to efficiently suppress specific genes or clusters from a genome.

### 8.2. Generate single-gene mutants to elucidate stress-alleviating mechanisms

Many studies characterize their strains of interest in terms of production of phytohormones, biofilm, siderophores, antibiotics, or phosphate solubilization capacity, and then try to correlate these traits with the stress-alleviating effects observed in plants when inoculating these strains. While this approach is interesting to describe new promising strains and to suggest the mechanisms potentially at play in stress alleviation, it does not provide conclusive evidence on the exact mechanisms involved. Yet, this understanding is central to identify and characterize bacterial functions of interest to help crops face climate-related stresses. Generating mutants for each of the traits potentially involved in stress alleviation would certainly help.

### 8.3. Screen for stress-tolerant strains alone

To improve plant growth under climate-related stresses, *Pseudomonas* strains must be able to cope with osmotic, ionic, heat, or flooding stress before helping the plant alleviate them. For instance, Yasmin et al. screened 44 bacterial strains for halotolerance and found a single *Pseudomonas* strain, *P. pseudoalcaligenes* SRM-16, that was able to survive under 20% NaCl conditions (Yasmin et al., 2020). This strain was subsequently shown to improve soybean tolerance to high salinity conditions. It is therefore important to assess the ability of a strain of interest to grow under stress conditions before testing it *in planta*.

### 8.4. Assess promising strains *in vivo* under different types of stresses

Many *Pseudomonas* strains have been shown to be effective in alleviating a specific climate-related stress in plants. In this review, we highlighted that some of these stresses, such as drought and high salinity, can affect plants in similar ways. *Pseudomonas* strains effective against a given stress may then be effective against others as well. We suggest assessing the effects of promising strains on multiple stresses rather than solely focusing on one. This could also offer fruitful collaboration opportunities between research groups working on different stresses.

### 8.5. Investigate how beneficial bacteria can help plants recover from stress

Research on plant stress alleviation by *Pseudomonas* strains often focuses on direct stress-alleviating effects. However, in the field, stress conditions are likely to last only for a limited period during the growing cycle, leaving time for the plants to recover. It would then be interesting to investigate the effect of beneficial bacteria on this recovery phase as well (Jagadish et al., 2021).

## Author contributions

AZ: conceptualization, investigation, writing—original draft, and writing—review and editing. MF: funding acquisition, supervision, and writing—review and editing. All authors contributed to the article and approved the submitted version.

## Funding

This work was supported by Agriculture and Agri-Food Canada under grants J-002366, J-002500, and J-002700.

## Conflict of interest

The authors declare that the research was conducted in the absence of any commercial or financial relationships that could be construed as a potential conflict of interest.

## Publisher’s note

All claims expressed in this article are solely those of the authors and do not necessarily represent those of their affiliated organizations, or those of the publisher, the editors and the reviewers. Any product that may be evaluated in this article, or claim that may be made by its manufacturer, is not guaranteed or endorsed by the publisher.
